# Predictors of intensive care unit admission in patients with hematologic malignancy

**DOI:** 10.1038/s41598-020-78114-7

**Published:** 2020-12-03

**Authors:** Abi Vijenthira, Nicholas Chiu, Daniel Jacobson, Zoey Freedman, Matthew C. Cheung, Shannon Goddard, Robert Fowler, Rena Buckstein

**Affiliations:** 1 Division of Medical Oncology and Hematology, Princess Margaret Cancer Centre, Toronto, Canada; 2 Boston University School of Medicine, Harvard T.H. Chan School of Public Health, Boston, USA; 3 Western University and Ivey Business School, London, Canada; 4 Postgraduate Genetics and Genome Sciences Education, Case Western Reserve University, Cleveland, USA; 5 Evaluative Clinical Sciences, Odette Cancer Research Program, Sunnybrook Research Institute, Toronto, Canada; 6 Department of Medicine, University of Toronto, Toronto, Canada; 7 Division of Medical Oncology and Hematology, Odette Cancer Centre, Sunnybrook Health Sciences Centre, Toronto, Canada; 8 Evaluative Clinical Sciences, Trauma, Emergency and Critical Care Research Program, Sunnybrook Research Institute, Toronto, Canada; 9 Sunnybrook Health Sciences Centre, Toronto, Canada; 10 Division of Critical Care Medicine, Department of Medicine and Interdepartmental, University of Toronto, Toronto, Canada; 11 Odette Cancer Research Program, Sunnybrook Research Institute, Toronto, Canada

**Keywords:** Haematological cancer, Cancer, Health care, Oncology

## Abstract

Limited data exist on predictors of intensive care unit (ICU) admission in patients with hematologic malignancy. The objective of this study was to identify predictors of ICU admission in hospitalized patients with hematologic malignancies. A retrospective cohort study was conducted on 820 consecutive admissions of patients with a malignant hematology diagnosis at our institution between March 2009 and December 2015. Backward stepwise selection procedure was conducted for multivariable logistic regression analyses. 820 patients were included, of whom 179 (22%) were admitted to the ICU. Types of hematologic cancers included 71% (N = 578) lymphoid cancer, 18% (N = 151) myeloid cancer, and 10% (N = 80) plasma cell neoplasms. 14% (N = 111) of patients had acute leukemia. Six predictors of admission to ICU were found in multivariable analysis, including disease-related (acute leukemia, curative intent chemotherapy), laboratory-related (platelet count < 50 × 109/L, albumin below normal, LDH above normal at time of admission), and physician-related factors (having advanced directives discussion) (*p* < 0.0001). A significant proportion of patients with hematologic malignancies admitted to hospital are admitted to ICU. Utilizing the identified predictors of ICU admission may help guide timely informed goals of care discussions with patients before clinical deterioration occurs.

## Introduction

The survival of patients with hematologic malignancies has improved rapidly over the past several decades, owing to advances in diagnosis, therapy, and supportive care. However, the prognosis of patients admitted to the intensive care unit (ICU) with hematologic malignancies remains guarded, with contemporary data showing up to 46%-90% mortality in the in-hospital setting^1–7^, which is significantly higher than the mortality of contemporaneous general medical patients admitted to the ICU^7^. Predictors of ICU mortality have been highlighted in several papers, and include need for vasopressors, mechanical ventilation, and severity of illness^1–6^.

However, limited data exist on predictors of ICU admission in patients with hematologic malignancy. Previous studies have been limited by both substantial heterogeneity in patient populations—combining a majority of patients with solid tumors with lesser numbers of patients with hematologic malignancies^8^—or substantial specificity, restricting to patients with a particular hematologic diagnosis^9^.

The primary objective of this study was to identify predictors of ICU admission in hospitalized patients with hematologic malignancies to enable more informed goals of care conversations with these patients on admission. The secondary objectives were to describe the clinical outcomes and predictors of outcomes among patients with hematologic malignancies who were admitted to hospital.

## Methods

A retrospective cohort study was conducted at a tertiary care centre in Toronto, Canada. Based on prior literature review, admission rate to ICU for patients with hematologic malignancies was estimated at 10%. Thus, to attain an event rate of at least 100 to allow exploration of up to 10–20 predictors of the outcome of ICU admission, we planned to examine 1000 consecutive patient admissions with a malignant hematology diagnosis at our institution, using hospital-level health records from March 19, 2009 to December 29, 2015. An additional 27 consecutive patients admitted to the ICU from March 19–July 29, 2009 were added to improve statistical power. At our institution, once a malignant hematology diagnosis is established, subsequent admissions to hospital occur under the malignant hematology service, with the exception of patients admitted for scheduled surgery. Such patients would typically be transferred to the malignant oncology service after the immediate post-operative period. Patients were included if they had any diagnosis of a hematologic malignancy, and were excluded if the sole a priori reason for admission was transfer to palliative care. For patients admitted more than once during this time period, only the first admission was included. As the rate of ICU admission was higher than anticipated, the final sample size was reduced. Chart review was conducted by two research students (DJ and NC) and audited by ZF and RB.

Variables were collected from review of electronic medical records and included: age, sex, body mass index, comorbidities, primary reason for admission, hematologic malignancy diagnosis, time from diagnosis to admission, all prior chemo- and/or immunotherapy treatments, documented intent of therapy: incurable (e.g. indolent lymphomas, myelodysplastic syndrome [MDS]), versus curative (e.g. acute myeloid leukemia [AML], acute lymphocytic leukemia [ALL], diffuse large B-cell lymphoma [DLBCL]), timing from last chemotherapy received and granulocyte-colony stimulating factor (G-CSF) use. We recorded laboratory values at time of admission including hemoglobin (HGB), platelet count (PLT), white blood cell count (WBC), absolute neutrophil count (ANC), absolute lymphocyte count (ALC), creatinine (Cr) and calculated creatinine clearance, liver enzymes (AST, ALT, ALP), total bilirubin, albumin, lactate dehydrogenase (LDH). We recorded whether there was documentation of an advanced directives discussion at any time during hospitalization, or directives to not perform cardiopulmonary resuscitation (CPR). Individual diagnoses and total score of the modified Charlson Comorbidity Index^10^ were recorded. The study was approved by the Sunnybrook research ethics board (#320-2017), and performed in accordance with the Tri-Council policies for ethical conduct in research. As this was a retrospective study, the need for informed consent was waived by the Sunnybrook research ethics board.

## Analysis

Demographic and clinical characteristics of all patients were described, including for those with and without ICU admission, using counts, proportions, medians, interquartile ranges (IQR) for categorical and continuous variables as appropriate. To compare factors between groups, Wilcoxon rank-sum nonparametric test or Fisher’s exact test were applied, as appropriate. To determine clinically important and statistically significant predictive factors of ICU admission and death in hospital or within 1 month of discharge, univariate logistic regression analysis was conducted. Because patients could be discharged or transferred to in-patient or home palliative care units, we categorized vital status as “death” for any death during hospitalization or until 30-days after hospital discharge. Natural log transformation was applied to certain covariates to normalize their distribution.

We used a backward stepwise selection procedure for multivariable logistic regression analyses and included any covariates with a *p* value of < 0.10 on univariate analysis. We considered several models with different covariates because of collinearity between these factors (e.g. PLT count < 50 or < 100 × 10^9^/L, total comorbidity score and individual comorbidities). The model with the highest R^2^ was selected as the final model. Kaplan–Meier survival curves were graphed and compared by the log-rank test. Bonferroni corrected *p* values < 0.002 (0.05/32 variables) were considered statistically significant for comparing the characteristics of patients admitted or not admitted to the ICU. *p* value of < 0.05 was considered statistically significant in the logistic regression analyses and log-rank test. All analyses were conducted using Statistical Analysis Software (SAS version 9.4 for Windows, Cary, NC) and R-package (version 3.5.0).

## Results

Data were collected on 820 patients, of whom 179 (22%) were admitted to the ICU. The median age was 66 years (IQR 56–76), and 44% were female (n = 358). The median time from diagnosis to hospital admission was 6.7 months (IQR 2.3–36.9), and the median time from last chemotherapy use was 1 month (IQR 0–3).

The types of hematologic malignancies included: (a) *lymphoid cancers, 578 patients (71%*): 52 patients (6%) with lymphoid very aggressive (ALL, lymphoblastic lymphoma, Burkitt lymphoma), 327 patients (40%) with lymphoid aggressive (e.g. DLBCL, peripheral T-cell lymphoma, anaplastic large cell lymphoma, primary central nervous system lymphoma etc.), and 199 patients (25%) with lymphoid indolent (follicular, marginal zone, small lymphocytic, etc.); (b)* myeloid cancers, 151 patients (18%)*: 87 patients (11%) with myeloid very aggressive (AML, blastic plasmacytoid dendritic cell neoplasm), 60 patients (7%) with myeloid aggressive (MDS, chronic myelomonocytic leukemia, etc.), and 4 patients (0.5%) with myeloid indolent (myeloproliferative neoplasms); (c)* plasma cell neoplasms, 80 patients (10%)*. Of the total sample of 820 patients, one hundred and eleven (14%) patients had acute leukemia and 45 (41%) were admitted to ICU. In contrast, only 21% (N = 78) and 24% (N = 19) of patients with very aggressive/aggressive lymphoma or plasma cell dyscrasias, respectively, were admitted to the ICU.

The most common causes for hospital admission were infections and febrile neutropenia (N = 289, 35%), followed by elective admission for chemotherapy (N = 139, 17%). Chemotherapy was administered to 262 patients (32%) during their admission. Further details on types of hematologic cancers and reasons for admission are depicted in Supplementary Table S1.

Seventy-three patients (41%) admitted to the ICU received mechanical ventilation, 17 (9%) received non-invasive ventilation, 8 (4%) received dialysis and 85 (47%) received vasoactive medications for blood pressure support. Eighty-four (47%) and 95 (53%) patients were admitted to high intensity and intermediate care units respectively.

### Timing and length of stay

The median length of stay in hospital was 7 days for all patients (IQR 4–15). The median time from hospitalization to ICU admission was 3 days (IQR 1–10, range 0–49) and the median ICU length of stay was 2 days (IQR 1–5, range 0–23). ICU admitted patients had longer total lengths of hospital stay than ward-alone patients (18 days (IQR 9–29) versus 5 days (IQR 3–10), *p* < 0.0001).

### Goals of care conversations

Among the 179 patients admitted to the ICU, 94 patients (53%) had a documented conversation about goals of care prior to ICU admission and of these, 76 patients (80%) chose full resuscitation in the event of cardiorespiratory failure. Among the 641 patients not admitted to the ICU, 208 patients (33%) had a goals of care conversation and of these, 110 patients (53%) chose full resuscitation.

### Characteristics of patients admitted to ICU

Patients admitted to the ICU had shorter times from cancer diagnosis, lower hemoglobin, platelet count, creatinine clearance and albumin, and higher LDH upon hospital admission compared to those who remained on the ward. They were more likely to be red blood cell transfusion dependent before ICU admission and during their hospital stay and to have received non-curative intent chemotherapy. A high proportion of ICU admitted patients (115 patients, 64%) had acute leukemia (myeloid predominated) or high-grade lymphomas. While not statistically significant after Bonferroni correction, ICU admitted patients had a higher proportion of elevated comorbidity scores (mCCI ≥ 2: 43% vs. 30%, *p* = 0.003). Infection as a cause of admission was not associated with ICU admission (Table 1).

**Table 1:** Demographic characteristics of patients.

	Total (N = 820)	Non-ICU admission (n = 641)	ICU admission (n = 179)	*p* value
**Age at admission (years)**				0.16
N	820	641	179	
Median (inter-quartiles)	66 (56, 76)	66 (56, 76)	64 (55, 74)	
Min, max	18, 98	18, 98	19, 96	
**BMI (kg/m**^**2**^**)**				0.58
N	709	577	132	
Median (inter-quartiles)	25.7 (22.1, 30.3)	25.7 (22.1, 30.6)	25.8 (22.0, 29.9)	
Min, max	7.3, 127.5	7.3, 127.5	14.7, 44.2	
**Gender**				0.23
Male	462 (56.3%)	354 (55.2%)	108 (60.3%)	
Female	358 (43.7%)	287 (44.8%)	71 (39.7%)	
**Time from diagnosis to admission (months)**				**0.0002**
N	819	640	179	
Median (inter-quartiles)	6.7 (2.3, 36.9)	7.0 (2.8, 41.3)	5.6 (0.8, 27.0)	
Min, max	0.0, 328.7	0.0, 328.7	0.0, 243.6	
**Time from last chemotherapy to admission (months)**				0.62
N	614	505	109	
Median (inter-quartiles)	1 (0, 3)	1 (0, 3)	1 (0, 4)	
Min, max	0, 232	0, 232	0, 113	
**Reason for admission (n = 819)**				0.02
Febrile neutropenia, infection, IP	289 (35.3%)	219 (34.2%)	70 (39.3%)	
Systemic (metabolic, CVS, HEME, RESP, GI, GU, Neuro, orthopaedic)	257 (31.4%)	201 (31.4%)	65 (36.3%)	
Directly cancer related (e.g. Chemotherapy, workup, ASCT, relapse)	184 (22.4%)	152 (23.7%)	32 (18.0%)	
Pain	33 (4.0%)	30 (4.7%)	3 (1.7%)	
FTT	56 (6.8%)	48 (7.5%)	8 (4.5%)	
**Any RBC transfusion preceding admission**				0.013
No	622 (75.9%)	499 (77.9%)	123 (68.7%)	
Yes	198 (24.2%)	142 (22.2%)	56 (31.3%)	
**PLT transfusion preceding admission**				0.58
No	733 (89.4%)	575 (89.7%)	158 (88.3%)	
Yes	87 (10.6%)	66 (10.3%)	21 (11.7%)	
**Chemotherapy intent**				**< .0001**
Curative	648 (79.0%)	529 (82.5%)	119 (66.5%)	
Non-curative	172 (21.0%)	112 (17.5%)	60 (33.5%)	
**Chemotherapy line**				0.26
N	641	506	135	
Median (inter-quartiles)	1 (1, 2)	1 (1, 2)	2 (1, 3)	
Min, max	1, 7	1, 6	1, 7	
**Chemotherapy pre-hospital admission**				0.31
No	179 (21.8%)	135 (21.1%)	44 (24.6%)	
Yes	641 (78.2%)	506 (78.9%)	135 (75.4%)	
**Acute leukemia**				**< .0001**
No	709 (86.5%)	575 (89.7%)	134 (74.9%)	
Yes	111 (13.5%)	66 (10.3%)	45 (25.1%)	
**Histology details (n = 809)**				**< .0001**
Lymphoid—very aggressive	52 (6.4%)	36 (5.7%)	16 (8.9%)	
Lymphoid—aggressive	327 (40.4%)	265 (42.1%)	62 (34.6%)	
Lymphoid—indolent	199 (24.6%)	166 (26.4%)	33 (18.4%)	
Myeloid—very aggressive	87 (10.8%)	50 (7.9%)	37 (20.7%)	
Myeloid—aggressive	60 (7.4%)	48 (7.6%)	12 (6.7%)	
Myeloid—indolent	4 (0.5%)	4 (0.6%)	0 (0%)	
Plasma cell neoplasm	80 (9.9%)	61 (9.7%)	19 (10.6%)	
**Laboratory values**				
**Hemoglobin**				**< .0001**
N	819	640	179	
Median (inter-quartiles)	95 (81, 113)	96 (83, 115)	89 (75, 106)	
Min, max	26, 161	26, 161	45, 152	
**Platelet count**				**< .0001**
N	817	640	177	
Median (inter-quartiles)	121 (43, 216)	135 (50, 224)	74 (22, 159)	
Min, max	1, 979	1, 979	2, 598	
**White blood cells**				0.46
N	820	641	179	
Median (inter-quartiles)	4.7 (1.7, 9.0)	4.6 (1.8, 8.6)	5.0 (1.5, 10.8)	
Min, max	0.0, 666.0	0.0, 553.4	0.0, 666.0	
**Creatinine clearance**				0.008
N	702	572	130	
Median (inter-quartiles)	78 (55, 111)	80 (57, 111)	69 (42, 104)	
Min, max	4, 352	11.4, 352.3	3.9, 226.2	
**Albumin**				** < .0001**
N	761	591	170	
Median (inter-quartiles)	36 (32, 40)	37 (33, 40)	33 (29, 39)	
Min, max	3, 51	3, 51	15, 47	
**LDH**				** < .0001**
N	497	416	81	
Median (inter-quartiles)	240 (182, 383)	231 (177, 346)	353 (206, 653)	
Min, max	0, 8175	0, 8175	0, 4500	
**Modified Charlson Comorbidity Index (CCI)**				
**Modified CCI**				0.02
N	820	641	179	
Median (inter-quartiles)	1 (0, 2)	1 (0, 2)	1 (0, 2)	
Min, max	0, 12	0, 12	0, 10	
**Congestive heart failure**				0.10
No	714 (87.1%)	565 (88.1%)	149 (83.2%)	
Yes	106 (12.9%)	76 (11.9%)	30 (16.8%)	
**PVD**				0.01
No	718 (87.6%)	571 (89.1%)	147 (82.1%)	
Yes	102 (12.4%)	70 (10.9%)	32 (17.9%)	
**Cerebrovascular disease**				0.09
No	774 (94.4%)	610 (95.2%)	164 (91.6%)	
Yes	46 (5.6%)	31 (4.8%)	15 (8.4%)	
**Dementia**				0.09
No	801 (97.7%)	623 (97.2%)	178 (99.4%)	
Yes	19 (2.3%)	18 (2.8%)	1 (0.6%)	
**Diabetes with end organ damage**				0.61
No	797 (97.2%)	624 (97.4%)	173 (96.6%)	
Yes	23 (2.8%)	17 (2.6%)	6 (3.4%)	
**Diabetes without end organ damage**				0.91
No	697 (85.0%)	544 (84.9%)	153 (85.5%)	
Yes	123 (15.0%)	97 (15.1%)	26 (14.5%)	
**Renal disease**				0.012
No	700 (85.4%)	558 (87.0%)	142 (79.3%)	
Yes	120 (14.6%)	83 (13.0%)	37 (20.7%)	
**Liver disease (mild)**				0.74
No	760 (92.7%)	595 (92.8%)	165 (92.2%)	
Yes	60 (7.3%)	46 (7.2%)	14 (7.8%)	
**Pulmonary disease**				0.02
No	764 (93.2%)	605 (94.4%)	159 (88.8%)	
Yes	56 (6.8%)	36 (5.6%)	20 (11.2%)	
**Solid cancer with metastases**				0.40
No	786 (95.9%)	612 (95.5%)	174 (97.2%)	
Yes	34 (4.2%)	29 (4.5%)	5 (2.8%)	
**Goals of care discussion in hospital pre-ICU**				** < .0001**
No	518 (63.2%)	433 (67.6%)	85 (47.5%)	
Yes	302 (36.8%)	208 (32.4%)	94 (52.5%)	
**Advance directive of not to attempt CPR**				0.089
No	704 (85.9%)	543 (84.7%)	161 (89.9%)	
Yes	116 (14.2%)	98 (15.3%)	18 (10.1%)	

*BMI* body mass index, *IP* infection-pneumonia, *HEME* hematologic, *RESP* respiratory, *GI* gastrointestinal, *GU* genitourinary, *ASCT* autologous stem cell transplant, *FTT* failure to thrive, *CVS* cardiovascular, *RBC* red blood cell *PVD* peripheral vascular disease, *ICU* intensive care unit, *CPR* cardiopulmonary resuscitation.

Bonferroni adjusted *p* value < 0.002 was considered statistically significant (bold).

The covariates associated with ICU admission by univariate analysis are depicted in Supplementary Table S2. By multivariable analysis, the covariates most associated with ICU admission included chemotherapy intent (non-curative vs. curative), PLT < 50 × 10^9^/L, acute leukemia, albumin below normal limits (< 37 g/L), LDH above normal limits (> 250 U/L), and advanced directive discussion in hospital pre-ICU admission (yes versus no) (Table 2). Age was not significant in univariate analysis and thus was not tested in the multivariable model.

**Table 2:** Multivariable logistic regression model of predictors of ICU admission.

Final model	*p* value	OR	95% CI of OR		Model fit R^2^ (%)
Intercept	< .0001				10.56
Chemotherapy intent (palliative vs. curative)	**0.02**	2.09	1.13	3.78	
Platelet count < 50 × 10^9^/L (yes vs. no)	**0.04**	1.76	1.00	3.06	
Acute leukemia (yes vs. no)	**0.0004**	3.32	1.70	6.39	
Albumin below normal (yes vs. no)	**0.01**	1.97	1.16	3.35	
LDH above normal (yes vs. no)	**0.02**	1.85	1.09	3.20	
Advanced directive discussion in hospital pre ICU (yes vs. no)	**0.01**	1.95	1.15	3.32	

### Overall survival

**Figure 1:**
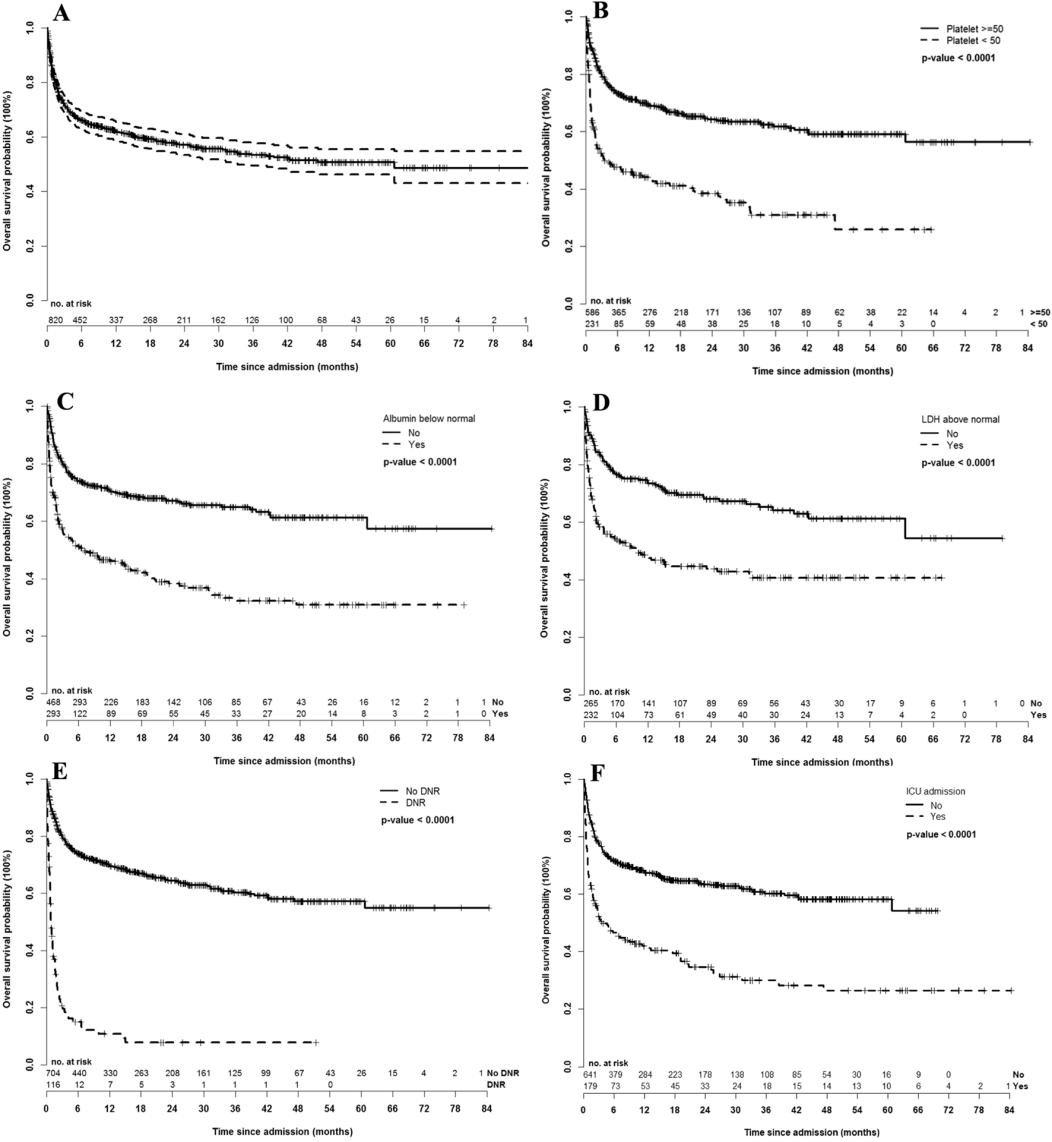
(**A**) Overall survival of all patients; (**B**) overall survival stratified by platelet count < 50 × 10^9^/L or ≥ 50 × 10^9^/L; (**C**) overall survival stratified by albumin above or below normal; (**D**) overall survival stratified by lactate dehydrogenase (LDH) above or below normal; (**E**) overall survival stratified by do not resuscitate (DNR) status; (**F**) overall survival stratified by intensive care unit admission (yes/no).

At a median follow-up of 8.1 months (IQR 1.5–25), a total of 328 patients (40%) died in the study, including 179 (22%) who died during hospitalization or within 1 month of discharge (Fig. 1). Of ICU admitted patients (n = 179), 43% had died within 1 month, including 37 (21%) who died in the ICU, 35 (19.6%) who died on the ward and 6 (3%) who died within 1 month of discharge. Median time to death after ICU admission was 4.5 days overall (IQR 1–14) but only 1 day for those who died in the ICU (IQR 0–2, range 0–8). Of non-ICU patients (n = 641), 16% had died within 1 month, including 45 (7%) who died on the ward and 56 (9%) who died within 1 month of discharge. Median time to death in non-ICU patients who died on the ward or within 1 month of discharge was 21 days (IQR 9–31) and 11 days (IQR 6–25) for death on the ward only. The patients admitted to the ICU had inferior overall survival with an actuarial survival of 3.7 months compared with not reached *p* < 0.0001 (Fig. 1F).

By univariate analysis, predictors of death in hospital or within 1 month of discharge for ICU admitted patients included admission to a high intensity (versus step-down) ICU (OR 2.4, *p* = 0.004), having had an advanced directives discussion (OR 1.9, *p* = 0.03); higher than normal values of WBC (OR 1.2, *p* = 0.04), ALP (OR 1.9, *p* = 0.02), LDH (OR 3.2, *p* = 0.01), receiving mechanical ventilation (OR 4.8, *p* < 0.0001) or vasopressors (OR 2.1, *p* = 0.02), and having a history of a solid tumor (OR 2.4, *p* = 0.03). Age, BMI, mCCI and creatinine clearance were not predictive.

By multivariable analysis, LDH exceeding normal (OR 3.6, *p* = 0.02) and receiving mechanical ventilation at any time during hospitalization (OR 5.4, *p* = 0.001) were the only measures independently predictive of death in ICU admitted patients.

When considering all patients admitted to hospital, the covariates predictive of death in hospital (in ICU or ward) or within 1 month of discharge by multivariable analysis were: age (OR 1.03 per year of age, *p* = 0.005), platelet count < 50 × 10^9^/L (OR 2.7, *p* = 0.0007), albumin below normal (OR 2.1, p = 0.01), LDH above normal (OR 3.9, *p* < 0.0001), an advanced directive of no CPR (OR 10.8, *p* < 0.0001) and having an ICU admission (OR 8.3, *p* < 0.0001) (Table 3 and Fig. 1).

**Table 3:** Multivariable logistic regression model of predictors of death during hospitalization or within 1 month of discharge in all hospitalized patients.

Final model	*p* value	OR	95% CI of OR		Model fit R^2^ (%)
Intercept	**< .0001**				31.25
Age at admission (years)	**0.004**	**1.03**	1.01	1.05	
Platelet count at admission < 50 × 10^9^/L (yes vs. no)	**0.0007**	**2.70**	1.52	4.82	
Albumin below normal at admission (yes vs. no)	**0.01**	**2.09**	1.18	3.70	
LDH above normal at admission (yes vs. no)	**< .0001**	**3.93**	2.19	7.28	
No cardiopulmonary resuscitation order documented at anytime during hospitalization (yes vs. no)	**< .0001**	**10.84**	5.46	22.16	
ICU admission (yes vs. no)	** < .0001**	**8.26**	4.30	16.21	

## Discussion

ICU morbidity and mortality remain an important issue for patients with hematologic malignancy. Although predictors of mortality in patients admitted to ICU with hematologic malignancy are well described^1,4–6,11–22^, there is a paucity of evidence that identifies the predictors of ICU admission in this population, or predictors of survival in hospitalized patients with hematologic malignancies. Overall, during a period of 5.5 years, 19% of patients with hematologic malignancies admitted to our tertiary care hospital were admitted to an ICU at least once, with 47% of these admissions to a high acuity (versus step-down) ICU.

Interestingly, we found that the predictors of ICU admission in hospitalized patients with hematologic cancers were a combination of disease-related (acute leukemia, curative intent chemotherapy), laboratory-related (platelet count, albumin, LDH), and physician-related factors (having advanced directives discussion). The latter factor likely represents the fact that physicians are more likely to have goals of care discussions in patients with impending or actual deterioration. The disease-related factors of acute leukemia and curative intent chemotherapy likely represent the more aggressive philosophy of care that patients and physicians have in diseases with curative potential.

It is noteworthy that ICU admission was independently associated with an eight times greater risk of death within 30 days compared to other admitted patients. Forty percent of ICU-admitted patients died in hospital, with patients presenting with an increased LDH and those requiring mechanical ventilation at greatest risk for death. The early and short-term mortality rates for ICU admitted patients were high (cumulative mortality rates at 1 month 34% and at 6 months 53%). This is similar to previous data, which report a range of in-hospital mortality of 46–90%^1,4–6,11–22^, with multi-organ dysfunction (illustrated by use of mechanical ventilation, vasopressor use, renal replacement therapy, significant liver enzyme elevation, high Acute Physiologic Assessment and Chronic Health Evaluation (APACHE) II, Sequential Organ Failure Assessment (SOFA), or Simplified Acute Physiology Score (SAPS) scores) being the most common predictor of death^1,4–6,11–22^. This was further confirmed in larger studies including a literature review of observational studies encompassing 10,000 patients^23^, and a retrospective analysis of 7689 patients with hematologic malignancy admitted to the ICU^24^. In fact, this is similar to the contemporary literature of predictive factors for mortality in general medical patients admitted to ICU^25^. However, this is the first time that LDH has been reported as an independent risk factor for mortality in patients with hematologic malignancy.

Notably, we did not find that body mass index, infection as a reason for admission, disease histology, presence of leukemia, recent chemotherapy, chemotherapy intent, line of chemotherapy, cytopenias, albumin, or comorbidity index score to be associated with an increased risk of death, which had been reported by others^17,23^. This may reflect the smaller sample size of ICU admitted patients in this analysis or the selection bias inherent with offering patients with acute leukemia induction chemotherapy. Of interest is one study that reported that the prognosis of a patient’s hematologic malignancy does not affect ICU mortality^26^, while others found that leukemia, recent transplant, Hodgkin lymphoma, or untreated malignancy were independent risk factors for mortality^3,27^; furthermore, hematologic malignancy is one variable of the SAPS score.

The cumulative mortality rate for non-ICU admitted patients was not low (1 month 12%, 6 months 28%) suggesting that any admission for toxicity or diagnosis related to a hematologic cancer is associated with inferior survival. Other factors independently predictive of death in hospital or within 1 month of discharge for all patients were: age, PLT count < 50 × 10^9^/L, reduced albumin, elevated LDH, and an advanced directive of no CPR. Apart from the advanced directives, these clinical and laboratory variables are valuable for counselling patients, families and health care providers especially since only 53% of ICU admitted and 33% of non-ICU admitted patients had advanced directives discussions documented in the charts.

There are several limitations to our study. This was a retrospective study, which may be limited by selection bias and incomplete data although sequential admissions were captured accurately by health data specialists. It is possible that advanced directive discussions occurred that were not documented in the electronic or paper patient chart, although this seems unlikely, and both were reviewed by chart audit. We did not record the timing of the advanced directives discussion and many may have occurred very near to clinical deterioration and potential death.

To be consistent among all patients, the laboratory values we considered were those upon admission to hospital and not during the hospital stay, which can increase or decrease with time and may be more frequently assayed in the sicker patients admitted to the ICU. Since admission to ICU from hospitalization was short, we believe there may still be some value in considering these admission laboratory parameters. We did not have APACHE, SOFA or SAPS scores to consider, which undoubtedly contributed to the paucity of predictors for mortality related to ICU admitted patients.

The strengths of our study include a large sample size of 870 patients with a heterogeneous collection of hematologic cancers and our unique focus on the predictors of ICU admission and overall survival in all patients admitted to hospital with hematologic cancers, which to our knowledge has never been reported before. As more patients with hematological malignancy are surviving and undergoing treatment, it becomes increasingly important to identify not only which patients will be admitted to ICU, but which patients may benefit from an intensive care unit admission.

The best discussions with patients about risk of mortality and morbidity occur outside of crisis situations. The predictive factors for ICU admission that we determined at our institution to be important at the time of hospitalization (non-curative intent chemotherapy, PLT < 50 × 10^9^/L, acute leukemia, low albumin, elevated LDH) may remind clinicians about the need for goals of care discussions before deterioration occurs. With the advent of big data, artificial intelligence and machine learning algorithms, one could envision a predictive score being generated in hospitalized patients, which could be used to guide clinical practice including the more careful monitoring of higher risk patients and complete documentation of goals of care.

## Supplementary information

Supplementary Tables.
