# Supplementary material for: Predictors of intensive care unit admission in patients with hematologic malignancy
Source: Sci Rep. 2020 Dec 3;10:21145. doi: 10.1038/s41598-020-78114-7 (PMC7713054; doi:10.1038/s41598-020-78114-7)
Supplement: Supplementary file 1 — Supplementary Tables. [file 41598_2020_78114_MOESM1_ESM.docx]

**Supplementary Material**

**Title:** Predictors of intensive care unit admission in patients with hematologic malignancy

**Authors:** Vijenthira A, Chiu N, Jacobson D, Freedman Z, Cheung MC, Goddard S, Fowler R, Buckstein R.

**Corresponding author:** Dr. Rena Buckstein (rena.buckstein@sunnybrook.ca)

| Table S1: Reasons for admission to hospital and subtypes of hematologic malignancies admitted to hospital | | | | | |
| --- | --- | --- | --- | --- | --- |
|  | **Total (N=820)** | | **Non-ICU Admission  (n=641)** | **ICU Admission (n=179)** | **p-value** |
| **Reason for admission** |  |  |  |  | 0.0231 |
| Anemia | 1 | (0.12%) | 0 (0.00%) | 1 (0.56%) |  |
| Autologous stem cell transplant | 3 | (0.37%) | 1 (0.16%) | 2 (1.12%) |  |
| Bleeding | 21 | (2.56%) | 14 (2.18%) | 7 (3.93%) |  |
| Bone | 1 | (0.12%) | 0 (0.00%) | 1 (0.56%) |  |
| Cardiovascular | 15 | (1.83%) | 13 (2.03%) | 2 (1.12%) |  |
| Edema | 7 | (0.85%) | 7 (1.09%) | 0 (0.00%) |  |
| Elective admission for chemotherapy | 139 | (16.97%) | 115 (17.94%) | 24 (13.48%) |  |
| Elective admission for procedure | 2 | (0.24%) | 2 (0.31%) | 0 (0.00%) |  |
| Elective admission for surgery | 22 | (2.69%) | 19 (2.96%) | 3 (1.69%) |  |
| Elective admission for therapy | 1 | (0.12%) | 0 (0.00%) | 1 (0.56%) |  |
| Elective admission for transfusion | 1 | (0.12%) | 1 (0.16%) | 0 (0.00%) |  |
| Elective admission for treatment | 9 | (1.10%) | 9 (1.40%) | 0 (0.00%) |  |
| Head and neck | 1 | (0.12%) | 1 (0.16%) | 0 (0.00%) |  |
| Febrile neutropenia | 132 | (16.12%) | 99 (15.44%) | 33 (18.54%) |  |
| Failure to thrive | 56 | (6.84%) | 48 (7.49%) | 8 (4.49%) |  |
| Gastrointestinal | 44 | (5.37%) | 34 (5.30%) | 10 (5.62%) |  |
| Genitourinary | 1 | (0.12%) | 1 (0.16%) | 0 (0.00%) |  |
| Hematologic | 36 | (4.40%) | 28 (4.37%) | 8 (4.49%) |  |
| Infection | 120 | (14.65%) | 95 (14.82%) | 25 (14.04%) |  |
| Pulmonary infection | 37 | (4.52%) | 25 (3.90%) | 12 (6.74%) |  |
| Metabolic | 15 | (1.83%) | 11 (1.72%) | 4 (2.25%) |  |
| Neurologic | 24 | (2.93%) | 16 (2.50%) | 8 (4.49%) |  |
| Pain | 33 | (4.03%) | 30 (4.68%) | 3 (1.69%) |  |
| Relapse | 3 | (0.37%) | 1 (0.16%) | 2 (1.12%) |  |
| Renal | 15 | (1.83%) | 9 (1.40%) | 6 (3.37%) |  |
| Respiratory | 72 | (8.79%) | 54 (8.42%) | 18 (10.11%) |  |
| Rheumatologic | 1 | (0.12%) | 1 (0.16%) | 0 (0.00%) |  |
| Swelling | 1 | (0.12%) | 1 (0.16%) | 0 (0.00%) |  |
| Thrombosis | 2 | (0.24%) | 2 (0.31%) | 0 (0.00%) |  |
| Workup | 4 | (0.49%) | 4 (0.62%) | 0 (0.00%) |  |
| **Disease** |  |  |  |  | 0.0099 |
| **Acute leukemias** |  |  |  |  |  |
| Acute lymphocytic leukemia | 26 | (3.17%) | 18 (2.81%) | 8 (4.47%) |  |
| Acute myeloid leukemia (AML) | 83 | (10.12%) | 48 (7.49%) | 35 (19.55%) |  |
| Acute promylocytic leukemia | 3 | (0.37%) | 2 (0.31%) | 1 (0.56%) |  |
| Prolymphocytic leukemia | 1 | (0.12%) | 0 (0.00%) | 1 (0.56%) |  |
| T-prolymphocytic leukemia | 1 | (0.12%) | 1 (0.16%) | 0 (0.00%) |  |
| **Lymphoid** |  |  |  |  |  |
| *Very aggressive* |  |  |  |  |  |
| Burkitts lymphoma | 24 | (2.93%) | 18 (2.81%) | 6 (3.35%) |  |
| Lymphoblastic lymphoma | 2 | (0.24%) | 0 (0.00%) | 2 (1.12%) |  |
| *Aggressive* |  |  |  |  |  |
| Anaplastic large cell lymphoma | 2 | (0.24%) | 2 (0.32%) | 0 (0.00%) |  |
| Acute T Cell Lymphoma (HTLV-1) | 2 | (0.24%) | 1 (0.16%) | 1 (0.56%) |  |
| Diffuse Large B cell lymphoma | 224 | (27.32%) | 183 (28.55%) | 41 (22.91%) |  |
| Hodgkins lymphoma | 29 | (3.53%) | 22 (3.43%) | 7 (3.91%) |  |
| Natural killer T cell lymphoma | 6 | (0.79%) | 4 (0.78%) | 1 (0.56%) |  |
| Peripheral T cell lymphoma | 38 | (4.63%) | 27 (4.21%) | 11 (6.15%) |  |
| Primary CNS lymphoma | 25 | (3.05%) | 24 (3.75%) | 1 (0.56%) |  |
| Sezary syndrome | 2 | (0.24%) | 2 (0.31%) | 0 (0.00%) |  |
| *Indolent* |  |  |  |  |  |
| B-cell lymphoma | 8 | (0.98%) | 8 (1.25%) | 0 (0.00%) |  |
| Chronic lymphocytic leukemia (CLL) | 74 | (9.02%) | 60 (9.36%) | 14 (7.82%) |  |
| Cutaneous T cell lymphoma | 2 | (0.24%) | 2 (0.31%) | 0 (0.00%) |  |
| Follicular lymphoma | 47 | (5.73%) | 39 (6.08%) | 8 (4.47%) |  |
| Hairy cell leukemia | 8 | (0.98%) | 5 (0.78%) | 3 (1.68%) |  |
| Large granular lymphocyte syndrome | 2 | (0.24%) | 2 (0.31%) | 0 (0.00%) |  |
| Lymphoplasmacytic lymphoma | 1 | (0.12%) | 1 (0.16%) | 0 (0.00%) |  |
| Mucosa associated lymphoid tissue lymphoma | 4 | (0.49%) | 4 (0.62%) | 0 (0.00%) |  |
| Mantle cell lymphoma | 23 | (2.80%) | 20 (3.12%) | 3 (1.68%) |  |
| Marginal zone lymphoma | 23 | (2.80%) | 19 (2.96%) | 4 (2.23%) |  |
| Small lymphocytic lymphoma | 1 | (0.12%) | 1 (0.16%) | 0 (0.00%) |  |
| Waldenstroms macroglobulinemia | 7 | (0.85%) | 7 (1.09%) | 0 (0.00%) |  |
| **Myeloid** |  |  |  |  |  |
| *Very aggressive* |  |  |  |  |  |
| Blastic plasmacytic dendritic cell neoplasm | 2 | (0.24%) | 1 (0.16%) | 1 (0.56%) |  |
| *Aggressive* |  |  |  |  |  |
| Chronic myelomonocytic leukemia | 3 | (0.37%) | 3 (0.47%) | 0 (0.00%) |  |
| Myelodysplastic syndrome (MDS) | 56 | (6.83%) | 45 (7.02%) | 11 (6.15%) |  |
| Myelodysplastic syndrome and CLL | 1 | (0.12%) | 1 (0.16%) | 0 (0.00%) |  |
| MDS / Multiple myeloma | 1 | (0.12%) | 1 (0.16%) | 0 (0.00%) |  |
| MDS/AML | 2 | (0.24%) | 1 (0.16%) | 1 (0.56%) |  |
| *Indolent* |  |  |  |  |  |
| Myelofibrosis | 4 | (0.49%) | 4 (0.62%) | 0 (0.00%) |  |
| **Plasma cell neoplasms** |  |  |  |  |  |
| Multiple myeloma | 81 | (9.88%) | 62 (9.68%) | 19 (10.61%) |  |
| **Other** | 1 | 0.12%) | 1 (0.16%) | 0 (0.00%) |  |
| ICU: intensive care unit; AML: acute myeloid leukemia; HTLV-1: Human T-lymphotrophic virus-1; CLL: chronic lymphocytic leukemia; MDS: myelodysplastic syndrome; CNS: central nervous system | | | | | |

| Table S2: Univariate Logistic Regression Analysis of ICU Admission | | | | | | |
| --- | --- | --- | --- | --- | --- | --- |
|  | Parameter Estimation | | | | Model Fitting Information R^2^ (%) | Included in the MVT |
| Independent variable | p-value | OR | 95% CI of OR | |  |  |
| ***Demographics*** |  |  |  |  |  |  |
| Age at admission (years) | 0.2874 | 0.994 | 0.984 | 1.005 | 0.14% |  |
| BMI (kg/m^2^) | 0.2054 | 0.982 | 0.954 | 1.007 | 0.26% |  |
| BSA | 0.4568 | 1.371 | 0.595 | 3.143 | 0.08% |  |
| Gender (F vs. M) | 0.2235 | 0.811 | 0.577 | 1.134 | 0.18% |  |
| **Time from diagnosis to admission (months) *** | **0.0004** | **0.811** | **0.721** | **0.908** | **1.61%** | **Y** |
| Time from last chemotherapy to admission (months) * | 0.6347 | 1.043 | 0.871 | 1.236 | 0.04% |  |
| Infection reason for admission (Infection vs. Other | 0.2029 | 1.249 | 0.885 | 1.755 | 0.20% |  |
| ***Clinical factors*** |  |  |  |  |  |  |
| **RBC transfusion preceding admission (Yes vs. No)** | **0.0120** | **1.600** | **1.104** | **2.301** | **0.74%** | **Y** |
| PLT transfusion preceding admission (Yes vs. No) | 0.5816 | 1.158 | 0.673 | 1.920 | 0.04% |  |
| RBC/PLT transfusion preceding ICU admission (Yes vs. No) | 0.6489 | 0.925 | 0.661 | 1.291 | 0.03% |  |
| **Chemotherapy intent (Palliative vs. Curative)** | **<.0001** | **2.381** | **1.638** | **3.446** | **2.42%** | **Y** |
| Chemotherapy line numbers (Continuous) | 0.1150 | 1.160 | 0.961 | 1.391 | 0.38% |  |
| Chemotherapy line ≥2 (Yes vs. No) | 0.5993 | 1.107 | 0.757 | 1.620 | 0.04% |  |
| **Chemotherapy line ≥3 (Yes vs. No)** | **0.0891** | **1.476** | **0.933** | **2.295** | **0.43%** | **Y** |
| Chemotherapy administration during hospital stay pre-ICU admission (Yes vs. No) | 0.6004 | 0.906 | 0.624 | 1.302 | 0.03% |  |
| **Disease histology (3 categories)** | **0.0024** |  |  |  | **1.42%** | **N** |
| Lymphoid vs. Plasma cell neoplasm | 0.3397 | 0.763 | 0.445 | 1.359 |  |  |
| Myeloid vs. Plasma cell neoplasm | 0.1690 | 1.542 | 0.842 | 2.908 |  |  |
| **Myeloid vs. Lymphoid** | **0.0005** | **2.021** | **1.357** | **3.011** |  |  |
| **T Cell vs. B Cell** | **0.0140** | **2.791** | **1.192** | **6.249** | **0.94%** | **Y** |
| **Acute leukemia (Yes vs. No)** | **<.0001** | **2.926** | **1.909** | **4.457** | **2.80%** | **Y** |
| **Advanced Directive Discussion In hospital pre ICU (Yes vs. No)** | **<.0001** | **2.302** | **1.645** | **3.228** | **2.83%** | **Y** |
| **DNR (Yes vs. No)** | **0.0780** | **0.619** | **0.353** | **1.031** | **0.41%** | **Y** |
| ***Lab findings at admission to hospital*** |  |  |  |  |  |  |
| **HGB** | **0.0002** | **0.986** | **0.979** | **0.993** | **1.70%** | **Y** |
| WBC * | 0.2684 | 1.082 | 0.939 | 1.243 | 0.15% |  |
| ANC * | 0.3982 | 1.089 | 0.893 | 1.327 | 0.09% |  |
| ALC * | 0.4065 | 1.067 | 0.909 | 1.237 | 0.08% |  |
| **PLT < 100 (Yes vs. No)** | **0.0001** | **1.923** | **1.375** | **2.698** | **1.78%** | **Y** |
| **PLT < 50 (Yes vs. No)** | **<.0001** | **2.355** | **1.660** | **3.334** | **2.74%** | **Y** |
| **Creatinine *** | **<.0001** | **2.147** | **1.558** | **2.971** | **2.66%** | **N** |
| **AST *** | **<.0001** | **1.660** | **1.291** | **2.152** | **2.11%** | **Y** |
| **ALT *** | **0.0263** | **1.282** | **1.027** | **1.596** | **0.64%** | **Y** |
| **Bilirubin *** | **0.0095** | **1.363** | **1.077** | **1.722** | **0.88%** | **Y** |
| ALP * | 0.1183 | 1.288 | 0.932 | 1.762 | 0.32% |  |
| **Albumin below normal (Yes vs. No)** | **<.0001** | **2.853** | **2.015** | **4.060** | **4.52%** | **Y** |
| **LDH above normal (Yes vs. No)** | **0.0007** | **2.351** | **1.444** | **3.892** | **2.38%** | **Y** |
| **Creatinine clearance categories** | **<.0001** |  |  |  | **2.70%** | **Y** |
| **<30 vs. ≥60** | **<.0001** | **3.824** | **2.098** | **6.908** |  |  |
| 30-59 vs. ≥60 | 0.8004 | 0.940 | 0.571 | 1.504 |  |  |
| **<30 vs. 30-59** | **<.0001** | **4.069** | **2.049** | **8.081** |  |  |
| ***Modified CCI*** |  |  |  |  |  |  |
| **Modified CCI** | **0.0103** |  |  |  | **1.09%** | **Y** |
| 1 vs. 0 | 0.9085 | 1.026 | 0.657 | 1.590 |  |  |
| **≥2 vs. 0** | **0.0060** | **1.706** | **1.167** | **2.504** |  |  |
| **≥2 vs. 1** | **0.0215** | **1.663** | **1.078** | **2.565** |  |  |
| **Myocardial Infarction (Yes vs. No)** | **0.0057** | **2.524** | **1.288** | **4.825** | **0.86%** | **Y** |
| **CHF (Yes vs. No)** | **0.0852** | **1.497** | **0.934** | **2.349** | **0.34%** | **Y** |
| **PVD (Yes vs. No)** | **0.0136** | **1.776** | **1.114** | **2.781** | **0.70%** | **Y** |
| **Cerebrovascular disease (Yes vs. No)** | **0.0720** | **1.800** | **0.925** | **3.360** | **0.37%** | **Y** |
| Dementia (Yes vs. No) | 0.1122 | 0.194 | 0.011 | 0.952 | 0.50% |  |
| Connective Tissue Damage (Yes vs. No) | 0.1251 | 0.723 | 0.472 | 1.083 | 0.30% |  |
| **Peptic Ulcer Disease (Yes vs. No)** | **0.0091** | **2.932** | **1.279** | **6.562** | **0.77%** | **Y** |
| Diabetes with end organ damage (Yes vs. No) | 0.6168 | 1.273 | 0.454 | 3.116 | 0.03% |  |
| Diabetes without end organ damage (Yes vs. No) | 0.8405 | 0.953 | 0.587 | 1.502 | 0.00% |  |
| **Renal disease (Yes vs. No)** | **0.0104** | **1.752** | **1.131** | **2.674** | **0.76%** | **Y** |
| Solid Cancer (mild) (Yes vs. No) | 0.2888 | 1.282 | 0.799 | 2.008 | 0.13% |  |
| AIDS (Yes vs. No) | 0.5398 | 1.443 | 0.392 | 4.369 | 0.04% |  |
| Liver disease (mild) (Yes vs. No) | 0.7696 | 1.097 | 0.569 | 1.995 | 0.01% |  |
| G-CSF use (Yes vs. No) | 0.5044 | 0.890 | 0.630 | 1.250 | 0.05% |  |
| **Pulmonary disease (Yes vs. No)** | **0.0106** | **2.114** | **1.172** | **3.716** | **0.74%** | **Y** |
| Solid Cancer with metastases (Yes vs. No) | 0.3091 | 0.606 | 0.204 | 1.461 | 0.14% |  |
| ICU: intensive care unit; MVT: multivariate analysis; BMI: body mass index; BSA: body surface area; RBC: red blood cell; PLT: platelet; DNR: do not resuscitate; HGB: hemoglobin; WBC: white blood cell; ANC: absolute neutrophil count; AST: aspartate aminotransferase; ALT: alanine transaminase; ALP: alkaline phosphatase; LDH: lactate dehydrogenase; CCI: Charlson Comorbidity Index; CHF: congestive heart failure; PVD: peripheral vascular disease; AIDS: acquired immunodeficiency syndrome; G-CSF: granulocyte-colony stimulating factor | | | | | | |
| * natural log-transformation was applied for normalizing distribution | | | | | | |
